# Long-term administration of melatonin attenuates neuroinflammation in the aged mouse brain

**DOI:** 10.17179/excli2017-654

**Published:** 2018-07-02

**Authors:** Kannika Permpoonputtana, Patlada Tangweerasing, Sujira Mukda, Parichart Boontem, Chutikorn Nopparat, Piyarat Govitrapong

**Affiliations:** 1 National Institute for Child and Family Development, Mahidol University, Thailand; 2 Research Center for Neuroscience, Institute of Molecular Biosciences, Mahidol University, Thailand; 3 Chulabhorn Graduate Institute, Chulabhorn Royal Academy, Thailand; 4 Department of Pharmacology, Faculty of Science, Mahidol University, Thailand

**Keywords:** aging, neuroinflammation, melatonin, pro-inflammatory cytokine, NFkappaB, BDNF

## Abstract

Aging is often accompanied by a decline in cognitive function in conjunction with a variety of neurobiological changes, including neuroinflammation. Melatonin is a key endogenous indoleamine secreted by the pineal gland that plays a crucial role in the regulation of circadian rhythms, is a potent free radical scavenger, has anti-inflammatory activity and serves numerous other functions. However, the role of melatonin in sterile inflammation in the brain has not been fully investigated. In the present study, we investigated the neuroinflammation status in aged mouse brains. The results showed that the protein levels of integrin αM (CD11b), glial fibrillary acidic protein (GFAP), the major pro-inflammatory cytokines (interleukin-1 beta [IL-1β], interleukin-6 [IL-6], and tumor necrosis factor alpha [TNF-α]) and phosphor-nuclear factor kappa B (pNFκB) were significantly increased, while N-methyl-D-aspartate (NMDA) receptor subunits NR2A and NR2B, Ca^2+^/calmodulin-dependent protein kinase II (CaMKII), and brain-derived neurotrophic factor (BDNF) were down-regulated in the hippocampus and prefrontal cortex (PFC) of 22-months-old (aged) mice compared with 2-months-old (young adult) mice. Melatonin was administered in the drinking water to a cohort of the aged mice at a dose of 10 mg/kg/day, beginning at an age of 16 months for 6 months. Our results revealed that melatonin significantly attenuated the alterations in these protein levels. The present study suggests an advantageous role for melatonin in anti-inflammation, and this may lead to the prevention of memory impairment in aging.

## Introduction

Increasing age is associated with structural and functional declines that lead to an increased risk of chronic diseases such as osteoporosis (Ginaldi et al., 2005[17]), cardiovascular disease (North and Sinclair, 2012[31]), and age-associated neuronal disorders such as mild cognitive impairment (MCI) (Guidi et al., 2006[19]) and Alzheimer's disease (AD) (Castegna et al., 2002[10]). Chronic low-grade inflammatory state in the aged brain produced an excessive level of pro-inflammatory cytokines. This state is termed “inflammaging”. The overproduction of pro-inflammatory cytokines in the aged brain without an injury or infection could be associated with the microglia number and activity increase in normal aging (Dilger and Johnson, 2008[15]). Astrocytes are regularly co-activated with microglial cells. The increase in cytokines also contributes to both excitotoxicity and microglia activation, by mechanisms that implicate impaired glutamate uptake, inflammatory signals, and/or oxidative stress (Morales et al., 2014[28]). Previous studies support that exaggerated production of pro-inflammatory cytokines, such as IL-1β, in aged rats leads to long-term memory impairment (Abraham and Johnson, 2009[1]; Barrientos et al., 2012[3]). Interleukin-1 beta (IL-1β) activated different signaling pathways then resulted in activating the cell death pathway (Barry et al., 2005[5]; Moore et al., 2007[27]). IL-1β also interrupted synaptic plasticity and long-term potentiation (LTP) expression by the production of free radicals (Bliss and Collingridge, 1993[7]).

N-acetyl-5-methoxytryptamine, regularly known as melatonin, plays an important role in controlling circadian rhythms and is a direct and an indirect mediator that can take on other tasks such as a free radical scavenger, an antioxidant, an anti-apoptotic and an anti-inflammatory (Hardeland, 2013[20]). Melatonin prevents inflammation by antagonizing both excitotoxicity and mitochondrial dysfunction (Hardeland, 2013[20]). A previous study has reported that melatonin can decrease the levels of activated microglia and reactive astrocytes, and down-regulate inflammatory mediators such as phosphorylated nuclear factor kappa B65 (p-NFĸB65), IL-1β, and tumor necrosis factor alpha (TNF-α) in an aging mouse model. Furthermore, the effects of melatonin on attenuating memory impairment have also been reported (Ali et al., 2015[2]; Mukda et al., 2016[29]). 

Our previous study showed that aged mice have significantly impaired spatial memory in the Morris water maze task. The long-term administration of melatonin restored spatial memory impairment (Mukda et al., 2016[29]). In addition, our recent study also showed that the levels of pro-inflammatory cytokines, which were increased after induction of senescence with a sublethal dose of H_2_O_2_, could be attenuated by melatonin in SH-SY5Y cells (Nopparat et al., 2017[30]). Understanding the role of melatonin in sterile inflammation in the brain should be strongly considered and further investigated. Therefore, in this study, the neuroinflammatory state was investigated in the hippocampus and prefrontal cortex (PFC) in aged mice, and the effect of melatonin on the neuroinflammatory state was also investigated. 

## Material and Methods

### Chemicals and reagents

Mouse monoclonal anti-NFκB p65, mouse monoclonal anti-CaMKII, rabbit polyclonal anti-BDNF, and rabbit polyclonal anti-integrin αM antibodies were purchased from Santa Cruz Biotechnology, Inc. (Santa Cruz, CA, USA). Mouse monoclonal anti-actin, mouse monoclonal anti-NR2B and rabbit polyclonal anti-NR2A antibodies were purchased from Millipore (Billerica, MA, USA). Mouse monoclonal anti-GFAP, rabbit monoclonal anti-phospho-NFκB p65, rabbit monoclonal anti-TNF-α, rabbit monoclonal anti-IL-6, mouse monoclonal anti-IL-1β, and horseradish peroxidase (HRP)-conjugated IgG antibodies were purchased from Cell Signaling Technology, Inc. (Danvers, MA, USA). ECL Western Blotting Reagents were purchased from GE Healthcare (Little Chalfont, Buckinghamshire, UK). Other chemicals were purchased from Sigma-Aldrich (St. Louis, MO, USA).

### Melatonin treatment protocol

The melatonin treatment was performed according to our previous study protocol (Jenwitheesuk et al., 2017[22]; Mukda et al., 2016[29]). The ICR mice used in this study were obtained from the Mahidol University Animals Center, Thailand. The experimental protocol was approved by the Animal Ethics Committee in accordance with the guide for the care and use of laboratory animals prepared by Mahidol University. They were held in standard laboratory cages with food and water provided ad libitum and a 12 h light/dark cycle. They were randomly divided into three groups, including the young control (2 months old), aged (22 months old) and melatonin-pretreated aged mice. Mice in the melatonin-treated group were given melatonin for 6 months (started from 16-22 months old). Melatonin was freshly prepared daily with water to a final concentration of 20 mg/l. The weight and the consumption of water containing melatonin were measured in each mouse. The daily melatonin intake for each mouse was approximately 10 mg/kg of body weight. Vehicle control mice were given tap water as drinking water. Mice were sacrificed. The brains were immediately removed, hippocampus and prefrontal cortex were dissected and stored at −80 °C until use.

### Western blotting

We performed protein extraction and Western blot analyses as described previously (Jenwitheesuk et al., 2017[22]; Mukda et al., 2016[29]). The hippocampus and prefrontal cortex samples were lysed for 10 min in lysis buffer and then centrifuged. The protein concentration was determined by the Bradford method (Bradford, 1976[8]). Lysate proteins were denatured at 95 °C for 5 min in sample buffer. Proteins were separated by sodium dodecyl sulfate-polyacrylamide gel electrophoresis and transferred to a polyvinylidenedifluoride membrane. The membrane was then incubated with different primary antibodies, at 4 °C for 24 hrs. After washing, the membrane was incubated with HRP-conjugated horse anti-mouse or anti-rabbit IgG at room temperature. Finally, protein bands were visualized by enhanced chemiluminescence using ECL PlusTM Western blotting detection reagents. The immunoblot bands were quantified for the detection of densitometry.

### Statistical analysis

One-way analysis of variance (ANOVA) and Tukey's post hoc tests were used in this study to examine differences in the data. A *p v*alue less than 0.05 was considered statistically significant.

## Results

### Melatonin attenuated aging-induced gliosis

Numerous studies have indicated that old age results in an increase in microgliosis and astrocytosis (Ojo et al., 2015[34]). The effect of melatonin on aging-induced inflammatory conditions was determined using protein expression levels of CD11b and GFAP. These are specific markers of activated microglia and astrocyte cells, respectively.

**Figure 1:**
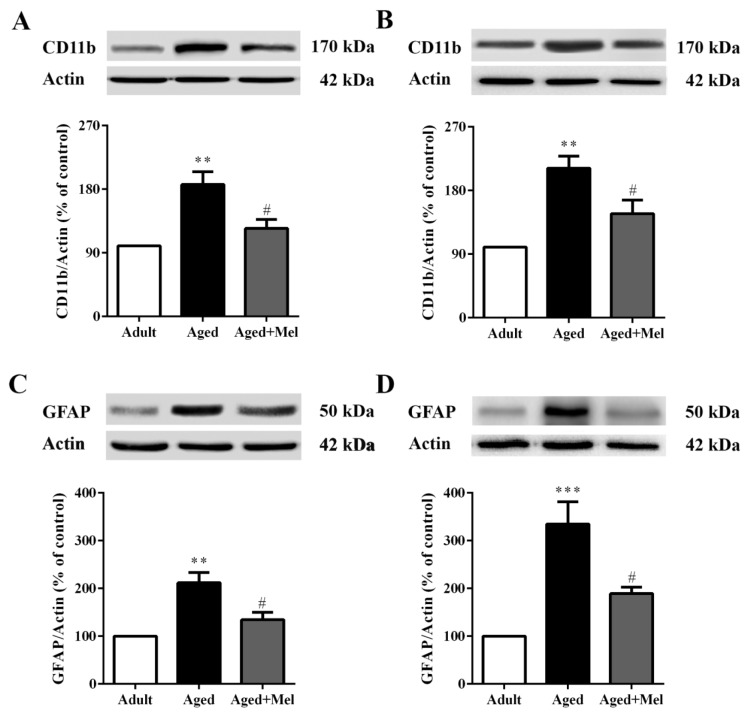
The effect of melatonin on CD11b and GFAP protein levels in the hippocampus (A, C) and the PFC (B, D) of aged mice. Mice were divided into three groups including the young control (2 months old), aged (22 months old) and melatonin-pretreated (10 mg/kg daily melatonin started from 16 to 22 months old) mice. Mice were sacrificed, brains were removed, hippocampus and prefrontal cortex were dissected and kept at -80 °C until use. The protein levels of CD11b and GFAP were determined by Western blot analyses. Representative bands from different groups are shown. The band densities were normalized to actin. The ratios were calculated as a percentage of the respective value of the control group. A one-way ANOVA was performed for statistical analysis. Data represent the mean ± S.E.M. from 4 mice. ** *p* < 0.01 and ***p < 0.001 compared with young adult mice, and ^#^*p *< 0.05 compared with aged mice

The Western blot analyses showed that CD11b protein expression (Supplementary Table 1) was significantly increased in the hippocampus (Figure 1A(Fig. 1)) and the PFC (Figure 1B(Fig. 1)) of aged mice compared to young adult mice. The GFAP protein expressions in the hippocampus (Figure 1C(Fig. 1)) and the PFC (Figure 1D(Fig. 1)) were significantly increased in aged mice compared with young adult mice. CD11b protein expressions in the hippocampus (Figure 1A(Fig. 1)) and the PFC (Figure 1B(Fig. 1)) were significantly reduced in melatonin-treated aged mice compared to the untreated aged mice. Accordingly, GFAP protein expressions in the hippocampus (Figure 1C(Fig. 1)) and the PFC (Figure 1D(Fig. 1)) were significantly decreased in melatonin-treated aged mice compared to untreated aged mice.

### Melatonin attenuated aging-induced increases in expression levels of pro-inflammatory cytokines 

Chronically activated microglia and astrocyte cells in aging display an increased production of pro-inflammatory cytokines that represent the most important causes of chronic inflammation. We examined major pro-inflammatory mediators including IL-1β, IL-6, and TNF-α.

**Figure 2:**
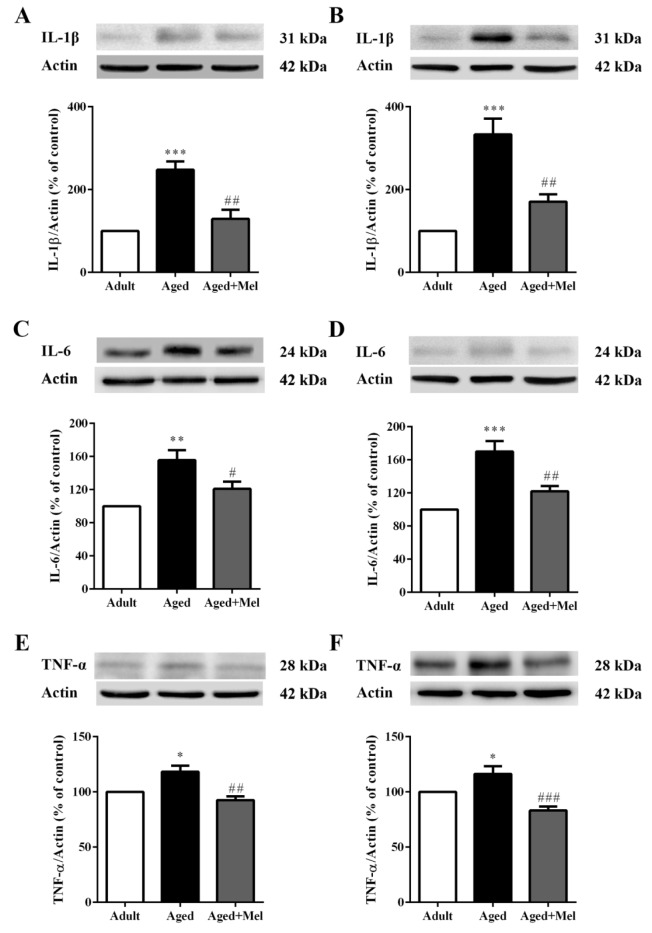
The effect of melatonin on IL-1β, IL-6, and TNF-α protein levels in the hippocampus (A, C, E) and the PFC (B, D, F) of aged mice. Mice were divided into three groups including the young control (2 months old), aged (22 months old) and melatonin-pretreated (10 mg/kg daily melatonin started from 16 to 22 months old) mice. Mice were sacrificed, brains were removed, hippocampus and prefrontal cortex were dissected and kept at -80 °C until use. The protein levels of IL-1β, IL-6, and TNF-α were determined by Western blot analyses. Representative bands from different groups are shown. The band densities were normalized to actin. The ratios were calculated as a percentage of the respective value of the control group. A one-way ANOVA was performed for statistical analysis. Data represent the mean ± S.E.M. from 4 mice. *p < 0.05, **p < 0.01 and ***p < 0.001 compared with young adult mice, and #p < 0.05, ##p < 0.01 and ###p < 0.001 compared with aged mice.

The Western blot analyses showed that IL-1β, IL-6, and TNF-α protein expression (Supplementary Table 2) in aged mice was significantly increased in the hippocampus (Figures 2A, 2C, and 2E, respectively(Fig. 2)) compared to the young adult mice. Additionally, IL-1β, IL-6, and TNF-α protein expressions in aged mice was significantly increased in the PFC (Figures 2B, 2D, and 2F, respectively(Fig. 2)) compared with young adult mice. Long-term administration of melatonin significantly decreased IL-1β, IL-6, and TNF-α protein expression in the hippocampus (Figures 2A, 2C, and 2E, respectively(Fig. 2)) compared to the aged mice without melatonin. Aged mice treated with melatonin showed significantly decrease in IL-1β, IL-6, and TNF-α protein expressions in the PFC (Figures 2B, 2D, and 2F, respectively(Fig. 2)) compared to the untreated aged mice.

### Melatonin attenuated aged-induced alterations in phosphorylated nuclear factor-kappa B (pNFκB) expression

**Figure 3:**
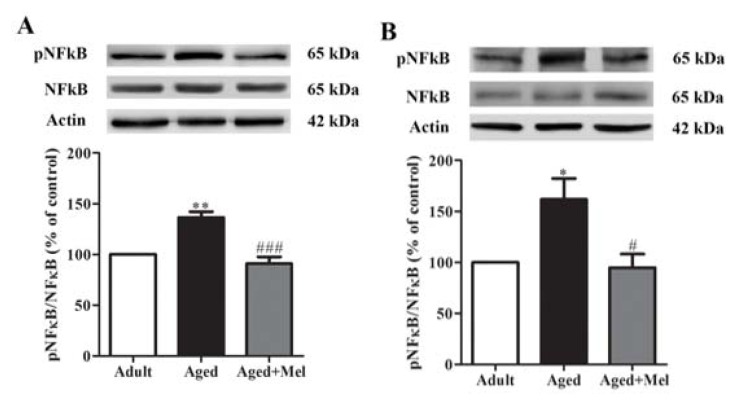
The effect of melatonin on pNFκB protein levels in the hippocampus (A) and the PFC (B) of aged mice. Mice were divided into three groups including the young control (2 months old), aged (22 months old) and melatonin-pretreated (10 mg/kg daily melatonin started from 16 to 22 months old) mice. Mice were sacrificed, brains were removed, hippocampus and prefrontal cortex were dissected and kept at -80 °C use. The protein levels of pNFκB and NFκB were determined by Western blot analyses. Representative bands from different groups are shown. The band densities were normalized to actin. The ratios were calculated as a percentage of the respective value of the control group. A one-way ANOVA was performed for statistical analysis. Data represent the mean ± S.E.M. from 4 mice. *p < 0.05 and **p < 0.01 compared with young adult mice, and #p < 0.05 and ###p < 0.001 compared with aged mice.

To investigate the effect of melatonin on pNFκB protein expression (Supplementary Table 3) in the hippocampus and PFC, Western blot analyses were performed. pNFκB protein expressions in aged mice were significantly increased in the hippocampus (Figure 3A(Fig. 3)) and the PFC (Figure 3B(Fig. 3)) compared to the young adult mice. Long-term administration of melatonin significantly decreased pNFκB protein expressions in the hippocampus (Figure 3A(Fig. 3)) and the PFC (Figure 3B(Fig. 3)) compared to the untreated aged mice.

### Melatonin treatment alleviated aged-induced alterations in NMDA receptors, CAMKII and BDNF

Aging is associated with the deterioration of memory function and changes in hippocampal function, including functional connectivity to the PFC (Tao et al., 2016[46]). Neuroinflammation plays an important role in the development of age-related memory impairment (Ali et al., 2015[2]). NMDA receptor subunits and the calcium/calmodulin-dependent protein kinase II (CaMKII) are necessary for LTP induction (Clayton et al., 2002[11]; Lisman et al., 2002[24]).

**Figure 4:**
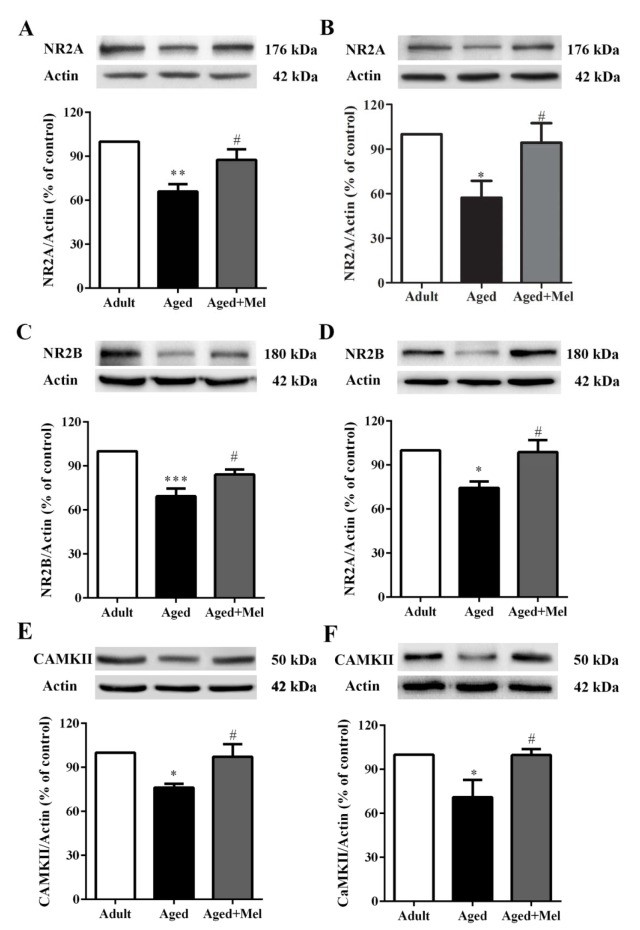
The effect of melatonin on NR2A, NR2B, and CaMKII protein levels in the hippocampus (A, C, E) and the PFC (B, D, F) of aged mice. Mice were divided into three groups including the young control (2 months old), aged (22 months old) and melatonin-pretreated (10 mg/kg daily melatonin started from 16 to 22 months old) mice. Mice were sacrificed, brains were removed, hippocampus and prefrontal cortex were dissected and kept at -80 °C until use. The protein levels of NR2A, NR2B, and CaMKII were determined by Western blot analyses. Representative bands from different groups are shown. The band densities were normalized to actin. The ratios were calculated as a percentage of the respective value of the control group. A one-way ANOVA was performed for statistical analysis. Data represent the mean ± S.E.M. from 4 mice. *p < 0.05, **p < 0.01 and ***p < 0.001 compared with young adult mice, and #p < 0.05 compared with aged mice.

The present results showed that in aged mice, NR2A protein expressions (Supplementary Table 4) were significantly decreased in the hippocampus (Figure 4A(Fig. 4)) and PFC (Figure 4B(Fig. 4)) compared with young adult mice. Melatonin-treated aged mice showed significantly increase in NR2A protein expressions in the hippocampus (Figure 4A(Fig. 4)) and PFC (Figure 4B(Fig. 4)) compared to the untreated aged mice. NR2B protein expressions in aged mice were significantly decreased in the hippocampus (Figure 4C(Fig. 4)) and PFC (Figure 4D(Fig. 4)) compared with young adult mice. Melatonin-treated aged mice showed significantly increase in NR2B protein expressions in the hippocampus (Figure 4C(Fig. 4)) and PFC (Figure 4D(Fig. 4)) compared with untreated aged mice. The CAMKII protein expressions in aged mice were significantly decreased in the hippocampus (Figure 4E(Fig. 4)) and PFC (Figure 4F(Fig. 4)) compared with young adult mice. Melatonin-treated aged mice showed significantly increase CAMKII protein expressions in the hippocampus (Figure 4E(Fig. 4)) and PFC (Figure 4F(Fig. 4)) compared to the untreated aged mice.

**Figure 5:**
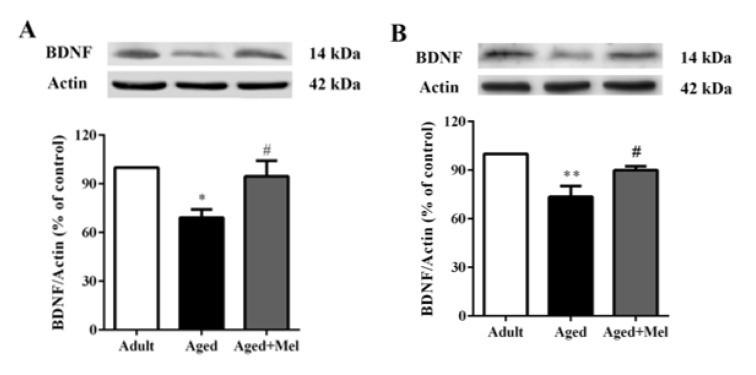
The effect of melatonin on BDNF protein levels in the hippocampus (A) and the PFC (B) of aged mice. Mice were divided into three groups including the young control (2 months old), aged (22 months old) and melatonin-pretreated (10 mg/kg daily melatonin started from 16 to 22 months old) mice. Mice were sacrificed, brains were removed, hippocampus and prefrontal cortex were dissected and kept at -80 °C until use. The protein levels of BDNF were determined by Western blot analyses. Representative bands from different groups are shown. The band densities were normalized to actin. The ratios were calculated as a percentage of the respective value of the control group. A one-way ANOVA was performed for statistical analysis. Data represent the mean ± S.E.M. from 4 mice. *p < 0.05, **p < 0.01 compared with young adult mice, and #p < 0.05 compared with aged mice.

An impairment in synaptic plasticity is not only represented by these outcomes showing an exaggerated neuroinflammatory response but also by a reduction in key downstream mediators such as brain-derived neurotrophic factor (BDNF) (Barrientos et al., 2004[4]). BDNF protein expressions (Supplementary Table 5) were significantly decreased in the hippocampus (Figure 5A(Fig. 5)) and PFC (Figure 5B(Fig. 5)) in aged mice compared with young adult mice. Melatonin-treated aged mice showed significantly increase in BDNF protein expressions in the hippocampus (Figure 5A(Fig. 5)) and PFC (Figure 5B(Fig. 5)) compared with untreated aged mice.

## Discussion

Our study in the hippocampus and PFC of aged mice showed that protein levels of microglia, astrocytes, and major pro-inflammatory cytokines including IL-1β, IL-6, and TNF-α were significantly increased compared with the levels in young adult mice. Melatonin administration significantly decreased these protein levels. In the young adult brain, normally quiescent microglia become activated microglia in response to a threat, with the result of anti-inflammatory cytokine production to facilitate a return to homeostasis (Colton, 2009[12]). In contrast, microglia in normal aging are characterized by an up-regulation in glial markers including the major histocompatibility complex class II (MHCII) and CD11b, a discovery that has been presented in various species such as canines, rodents, and non-human primates, as well as in human post-mortem tissue (Perry et al., 1993[37]; Rogers et al., 1988[39]), whereas MHCII is expressed at very low levels in the microglia of young animals (Perry, 1998[36]). A recent study found that CD11b and GFAP protein levels were elevated in aged rat brains, notably in the hippocampus (Ojo et al., 2011[33]) and in the PFC; the number of CD11b-positive cells in the aged mice was significantly larger than that in the young mice (Ohshima et al., 2015[32]).

The previous study indicated that inflammation in the aged brain is defined by up-regulated astrocytes and microglial cells coupled with increased levels of cytokines such as IL-1β, IL-6 and TNF-α (Ali et al., 2015[2]; Godbout and Johnson, 2004[18]). The elevation of NFĸB has been reported in old age (Calabrese et al., 2011[9]) and activation of NFκB, stimulation of other inflammatory mediators, and glial activation lead to progression of age-related diseases and other neurodegenerative diseases (Bierhaus et al., 2005[6]; Srikanth et al., 2011[45]).

The anti-inflammatory mechanism of melatonin is generally based on its antioxidant properties. Our previous study showed that the anti-neuroinflammatory effects of melatonin inhibited the activation of NF-κB and decreased TNF-α mRNA levels in methamphetamine-induced pro-inflammatory mediators (Permpoonputtana and Govitrapong, 2013[35]; Wongprayoon and Govitrapong, 2015[50]) and in the H_2_O_2_-induced senescence state (Nopparat et al., 2017[30]) of human neuroblastoma dopamine SH-SY5Y cell lines. Previous studies have established the ability of melatonin to alleviate inflammation in aged animal models. Melatonin has been shown to reduce pro-inflammatory cytokines and inducible nitric oxide synthase (iNOS) in the liver of aged rats (Kireev et al., 2008[23]). Moreover, melatonin can down-regulate the mRNA expression of IL-1β, TNF-α, NFκB and iNOS and the protein expression of IL-1β and TNF-α in the liver of SAMP8 mice (Cuesta et al., 2011[13]). In addition, melatonin plays an important role in inflammation in both pro-inflammatory and anti-inflammatory actions. A study by Cuesta et al. illustrated that IL-6 protein levels were decreased whereas IL-10 protein levels were increased in pancreatic cells of SAMP8 mice treated with melatonin (Cuesta et al., 2011[13]). Melatonin levels in mammals decline considerably with aging, resulting in numerous changes, especially adaptation of the immune system that can lead to various disorder and diseases (Hardeland et al., 2012[21]). 

Our previous study showed that melatonin attenuated the methamphetamine-induced TNFα overexpression and the NFκB activation via melatonin receptors, since the attenuating effect was prevented by preincubation with luzindole (an antagonist of melatonin receptors) and MT2 knockdown by siRNA (Wongprayoon and Govitrapong, 2015[50]). Melatonin administration was able to restore this reduction. A previous study (Sanchez-Hidalgo et al., 2009[40]) found an age-related reduction in MT1 and MT2 mRNA expression levels in the spleen, liver, kidney, and heart of aged rats. Our previous study also showed that MT1 and MT2 receptor protein expression in the hippocampus in aged mice was significantly decreased (Jenwitheesuk et al., 2017[22]). While melatonin exerts actions on neurogenesis via membrane-bound receptors in the subventricular zone (Sotthibundhu et al., 2010[44]) and the rat hippocampus (Tocharus et al., 2014[47]). 

The elevation of pNFĸB has been represented in a senescence model (Calabrese et al., 2011[9]; Nopparat et al., 2017[30]), and NFκB signaling has been shown to induce the production of pro-inflammatory cytokines leading to increasing age-related diseases such as memory impairment and AD (Ali et al., 2015[2]; Srikanth et al., 2011[45]). The N-methyl-D-aspartate receptor (NMDAR) subfamily of glutamate receptors is expressed in the CNS and is involved in higher brain functions such as memory formation and cognition (Rebola et al., 2010[38]). A previous study indicated that NMDAR function in neurons was regulated by local supporting cells, specifically microglia and astrocytes, which therefore provided an additional possible mechanism for the age-related changes in NMDAR function, such as the binding of D-serine, which comes from astrocytes as a main source (Schell et al., 1995[41]). D-serine decreased in the hippocampus and cortex of aged rats (Williams et al., 2006[49]). The present study found that NMDAR and CaMKII protein levels in the hippocampus and PFC were decreased in aged mice compared with young adult mice. A decrease in the NR2A protein expression in the hippocampus has been previously reported (Liu et al., 2008[25]; Sonntag et al., 2000[43]). The interaction between glutamate and NMDAR leads to the activation of several metabolic pathways such as CaMK, extracellular-signal-regulated kinases (ERK), and cAMP response element binding protein (CREB), which are responsible for the activation of LTP. CaMKII is a crucial protein for the induction of LTP (Lisman et al., 2002[24]) which can be prevented by a CaMKIIα knock-out or blocking agent (Malinow et al., 1989[26]; Silva et al., 1992[42]). 

The results of this exaggerated neuroinflammatory response include not only impairment in synaptic plasticity but also a reduction in key downstream mediators such as BDNF (Barrientos et al., 2004[4]). Moreover, in our study, BDNF protein levels in the hippocampus and PFC were significantly decreased in aged mice. BDNF, a member of the neurotrophin family, is not only involved in the differentiation and survival of neurons in the CNS but also an essential regulator of synaptogenesis and synaptic plasticity mechanisms underlying learning and memory in the adult CNS (Cunha et al., 2010[14]). The decrease of BDNF was correlated with a decline in hippocampal volume in the late adulthood (Erickson et al., 2010[16]; Ziegenhorn et al., 2007[51]). Our study showed that melatonin could increase the protein levels of NR2A, NR2B, CaMKII, and BDNF in aged mice. Melatonin has been found to enhance learning and memory function in our previous study and in studies by other investigators (Ali et al., 2015[2]; Mukda et al., 2016[29]; Tongjaroenbuangam et al., 2013[48]).

## Conclusion

In conclusion, we demonstrated that aging in mice was associated with the induction of inflammation, while melatonin reversed the increase in the neuroinflammatory state and rescued the decrease in NR2A, NR2B, CaMKII, and BDNF expression in the hippocampus and PFC in aged mice. These brain areas are related with learning and memory processes. Thus, the present study supports the advantageous roles of melatonin in regard to anti-aging through anti-inflammation and potential prevention of the risk of memory impairment. 

## Acknowledgements

The present study was supported by a research grant from Thailand Research Fund (DPG5780001) to PG and a Mahidol University Research Grant to PG and KP.

## Conflict of interest

The authors have no conflict of interest.

## Supplementary Material

Supplementary data
