# Supplementary material for: Long-term administration of melatonin attenuates neuroinflammation in the aged mouse brain
Source: EXCLI J. 2018 Jul 2;17:634–46. doi: 10.17179/excli2017-654 (PMC6088215; doi:10.17179/excli2017-654)
Supplement: Supplementary data [file EXCLI-17-634-s-001.pdf]

EXCLI Journal 2018;17:634-646 – ISSN 1611-2156
Received: July 21, 2017, accepted: June 11, 2018, published: July 02, 2018

Supplementary data to:

LONG-TERM ADMINISTRATION OF MELATONIN ATTENUATES NEUROINFLAMMATION IN THE AGED MOUSE BRAIN

Kannika Permpoonputtana[1], Patlada Tangweerasing[2], Sujira Mukda[2], Parichart Boontem[3], Chutikorn Nopparat[2], Piyarat Govitrapong[2,3,4,*]

[1] National Institute for Child and Family Development, Mahidol University, Thailand
[2] Research Center for Neuroscience, Institute of Molecular Biosciences, Mahidol University, Thailand
[3] Chulabhorn Graduate Institute, Chulabhorn Royal Academy, Thailand
[4] Department of Pharmacology, Faculty of Science, Mahidol University, Thailand

* Corresponding author: Piyarat Govitrapong, Chulabhorn Graduate Institute, Chulabhorn Royal Academy, 54 Kamphaeng Phet 6 Road, Lak Si, Bangkok 10210, Thailand,
E-mail: piyarat.gov@mahidol.ac.th, piyarat@cgi.ac.th

http://dx.doi.org/10.17179/excli2017-654

This is an Open Access article distributed under the terms of the Creative Commons Attribution License (http://creativecommons.org/licenses/by/4.0/).

Supplementary Table 1: The effect of melatonin on CD11b and GFAP protein levels in the hippocampus and the prefrontal cortex of aged mice

Type of protein	Brain area	Number of animal	Animal group		
			Young adult mice	Aged mice	Melatonin-treated aged mice
**CD11b**	**Hippocampus**	N_1	100	169.9	114.5
		N_2	100	158.4	95.9
		N_3	100	240.3	153.9
		N_4	100	177.6	134.3
		**Mean±S.E.M.**	**100**	**186.7±18.3****	**124.7±12.5#**
	**Prefrontal cortex**	N_1	100	259.1	187.8
		N_2	100	195.1	154.3
		N_3	100	182.4	94.3
		N_4	100	210.1	152.6
		**Mean±S.E.M.**	**100**	**211.7±16.8****	**147.3±19.4#**
**GFAP**	**Hippocampus**	N_1	100	171.6	101.6
		N_2	100	208.3	170.0
		N_3	100	272.4	150.2
		N_4	100	194.8	116.4
		**Mean±S.E.M.**	**100**	**211.8±21.6****	**134.6±15.6#**
	**Prefrontal cortex**	N_1	100	232.6	176.8
		N_2	100	281.4	210.4
		N_3	100	429.9	213.2
		N_4	100	395.9	156.1
		**Mean±S.E.M.**	**100**	**335.0±46.6*****	**189.1±13.8#**

CD11b and GFAP protein levels were determined by Western blot analyses. A one-way ANOVA was performed for statistical analysis. Data represent the mean ± S.E.M. from 4 mice. ** $p < 0.01$ and *** $p < 0.001$ compared with young adult mice, and $^{\#}p < 0.05$ compared with aged mice

EXCLI Journal 2018;17:634-646 – ISSN 1611-2156
Received: July 21, 2017, accepted: June 11, 2018, published: July 02, 2018

Supplementary Table 2: The effect of melatonin on IL-1β, IL-6, and TNF-α protein levels in the hippocampus and the prefrontal cortex of aged mice

Type of protein	Brain area	Number of animal	Animal group		
			Young adult mice	Aged mice	Melatonin-treated aged mice
**IL-1β**	**Hippocampus**	N_1	100	264.6	179.4
		N_2	100	292.2	152.8
		N_3	100	236.9	125.1
		N_4	100	197.4	99.0
		**Mean±S.E.M.**	**100**	**247.8±20.2*** **	**139.1±17.4$^{\#\#}$**
	**Prefrontal cortex**	N_1	100	249.6	208.4
		N_2	100	305.6	124.1
		N_3	100	430.8	180.5
		N_4	100	346.7	170.7
		**Mean±S.E.M.**	**100**	**333.2±38.1*** **	**170.9±17.5$^{\#\#}$**
**IL-6**	**Hippocampus**	N_1	100	165.3	111.9
		N_2	100	126.9	104.1
		N_3	100	182.8	143.8
		N_4	100	147.6	123.7
		**Mean±S.E.M.**	**100**	**155.7±12.0** **	**120.9±8.6$^{\#}$**
	**Prefrontal cortex**	N_1	100	185.5	105.4
		N_2	100	149.8	130.0
		N_3	100	147.9	132.7
		N_4	100	196.9	120.2
		**Mean±S.E.M.**	**100**	**170.0±12.5*** **	**122.1±6.2$^{\#\#}$**
**TNF-α**	**Hippocampus**	N_1	100	131.5	82.9
		N_2	100	115.5	94.4
		N_3	100	121.0	100.3
		N_4	100	105.5	92.5
		**Mean±S.E.M.**	**100**	**118.4±5.4***	**92.5±3.6$^{\#\#}$**
	**Prefrontal cortex**	N_1	100	108.4	83.6
		N_2	100	135.0	93.0
		N_3	100	120.3	80.5
		N_4	100	117.6	75.8
		**Mean±S.E.M.**	**100**	**120.3±5.5***	**83.2±3.6$^{\#\#\#}$**

IL-1β, IL-6, and TNF-α protein levels were determined by Western blot analyses. A one-way ANOVA was performed for statistical analysis. Data represent the mean ± S.E.M. from 4 mice. *$p < 0.05$, **$p < 0.01$ and ***$p < 0.001$ compared with young adult mice, and $^{\#}p < 0.05$, $^{\#\#}p < 0.01$ and $^{\#\#\#}p < 0.001$ compared with aged mice.

EXCLI Journal 2018;17:634-646 – ISSN 1611-2156
Received: July 21, 2017, accepted: June 11, 2018, published: July 02, 2018

Supplementary Table 3: The effect of melatonin on pNF-κB protein levels in the hippocampus and the and the prefrontal cortex of aged mice

Type of protein	Brain area	Number of animal	Animal group		
			Young adult mice	Aged mice	Melatonin-treated aged mice
**pNF-κB**	**Hippocampus**	N_1	100	131.0	88.0
		N_2	100	150.1	110.3
		N_3	100	140.4	86.7
		N_4	100	124.0	78.5
		**Mean±S.E.M.**	**100**	**136.4±5.7****	**90.9±6.8###**
	**Prefrontal cortex**	N_1	100	113.5	79.0
		N_2	100	170.4	82.7
		N_3	100	192.2	121.2
		N_4	100	122.5	84.0
		**Mean±S.E.M.**	**100**	**149.7±18.9***	**91.7±9.9#**

pNF-κB protein levels were determined by Western blot analyses. A one-way ANOVA was performed for statistical analysis. Data represent the mean ± S.E.M. from 4 mice. $^{*}p < 0.05$ and $^{**}p < 0.01$ compared with young adult mice, and $^{\#}p < 0.05$ and $^{\#\#\#}p < 0.001$ compared with aged mice.

Received: July 21, 2017, accepted: June 11, 2018, published: July 02, 2018

Supplementary Table 4: The effect of melatonin on NR2A, NR2B, and CaMKII protein levels in the hippocampus and the prefrontal cortex of aged mice

Type of protein	Brain area	Number of animal	Animal group		
			Young adult mice	Aged mice	Melatonin-treated aged mice
**NR2A**	**Hippocampus**	N_1	100	76.1	98.9
		N_2	100	53.3	66.6
		N_3	100	70.7	95.5
		N_4	100	64.4	89.5
		**Mean±S.E.M.**	**100**	**66.1±4.9****	**87.6±7.3$^{\#}$**
	**Prefrontal cortex**	N_1	100	78.2	120.5
		N_2	100	80.2	101.5
		N_3	100	61.7	91.2
		N_4	100	77.9	81.7
		**Mean±S.E.M.**	**100**	**74.5±4.3***	**98.7±8.3$^{\#}$**
**NR2B**	**Hippocampus**	N_1	100	84.5	79.9
		N_2	100	62.1	77.7
		N_3	100	66.6	86.8
		N_4	100	64.3	92.3
		**Mean±S.E.M.**	**100**	**69.4±5.1*****	**84.2±3.3$^{\#}$**
	**Prefrontal cortex**	N_1	100	74.5	97.0
		N_2	100	84.9	102.1
		N_3	100	65.9	81.3
		N_4	100	38.5	94.9
		**Mean±S.E.M.**	**100**	**66.0±9.9***	**93.8±4.4$^{\#}$**
**CaMKII**	**Hippocampus**	N_1	100	82.4	100.3
		N_2	100	70.6	120.1
		N_3	100	74.4	85.7
		N_4	100	77.7	83.0
		**Mean±S.E.M.**	**100**	**76.3±2.5***	**97.3±8.5$^{\#}$**
	**Prefrontal cortex**	N_1	100	53.4	88.2
		N_2	100	52.2	100.9
		N_3	100	101.5	107.0
		N_4	100	77.1	102.9
		**Mean±S.E.M.**	**100**	**71.1±11.7***	**99.8±4.1$^{\#}$**

NR2A, NR2B, and CaMKII protein levels were determined by Western blot analyses. A one-way ANOVA was performed for statistical analysis. Data represent the mean ± S.E.M. from 4 mice. *$p < 0.05$, **$p < 0.01$ and ***$p < 0.001$ compared with young adult mice, and $^{\#}p < 0.05$ compared with aged mice.

Supplementary Table 5: The effect of melatonin on BDNF protein levels in the hippocampus and the prefrontal cortex of aged mice

Type of protein	Brain area	Number of animal	Animal group		
			Young adult mice	Aged mice	Melatonin-treated aged mice
**BDNF**	**Hippocampus**	N_1	100	54.6	73.8
		N_2	100	73.5	108.2
		N_3	100	71.8	113.4
		N_4	100	77.1	82.7
		**Mean±S.E.M.**	**100**	**69.3±5.0***	**94.5±9.6$^{\#}$**
	**Prefrontal cortex**	N_1	100	54.9	95.7
		N_2	100	85.6	84.2
		N_3	100	74.1	87.5
		N_4	100	79.5	92.4
		**Mean±S.E.M.**	**100**	**73.5±6.6****	**90.0±2.5$^{\#}$**

BDNF protein levels were determined by Western blot analyses. A one-way ANOVA was performed for statistical analysis. Data represent the mean ± S.E.M. from 4 mice. *p < 0.05, **p < 0.01 compared with young adult mice, and $^{\#}p$ < 0.05 compared with aged mice
